# Supplementary material for: A Case of Phage Therapy against Pandrug-Resistant Achromobacter xylosoxidans in a 12-Year-Old Lung-Transplanted Cystic Fibrosis Patient
Source: Viruses. 2021 Jan 5;13(1):60. doi: 10.3390/v13010060 (PMC7824836; doi:10.3390/v13010060)
Supplement: Supplementary file 1 [file viruses-13-00060-s001.zip › Supplementary data/Supplementary Materials Phage Achromobacter Revision.docx]

**Supplementary Material**

**Supplementary Figure S1.** Antibiotic susceptibility testing (AST) of a pandrug-resistant *Achromobacter xylosoxidans* isolated during a bronchoalveolar lavage of a 12-year-old boy after double lung transplantation. AST was performed by antibiotic disk diffusion on Mueller-Hinton agar plate. A. Tested antibiotics (from top left to the right): TIC: ticarcillin; FEP: cefepime; PRL: piperacillin; CN: gentamicin; CAZ: ceftazidime; TIM: ticarcillin-clavulanic acid; CPO: cefpirome; TOB: tobramycin; SXT: trimethoprim-sulfamethoxazole; ATM: aztreonam; TPZ: piperacillin-tazobactam; AK: amikacin; CIP: ciprofloxacin; DOR: doripenem; MEM: meropenem; IPM: imipenem. B. Tested antibiotics (from top left, clockwise): FF: fosfomycin; RA: rifampin; LEV levofloxacin; TE: tetracycline; CT: chloramphenicol. Grey rectangles have been added to occult the patient’s name. This bacterial isolate was also resistant to ceftolozane/tazobactam, temocillin, colistin, tigecycline and imipenem with minimal inhibitory concentrations (MICs) of > 256, 1024, 256, 6 and 32 mg/L, respectively.


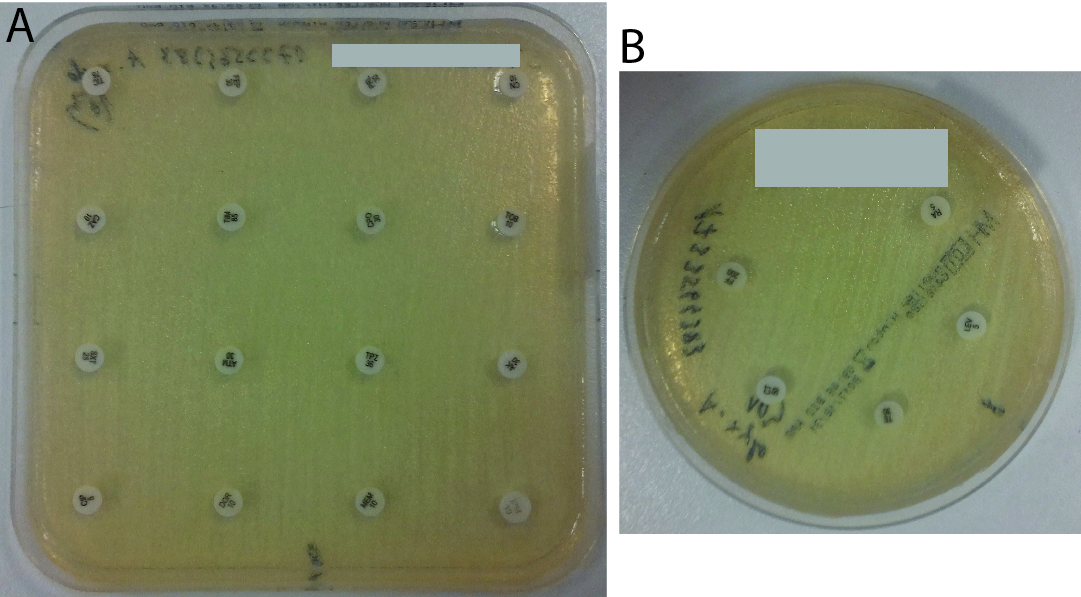


**Supplementary Tables**

**Supplementary Table S1**. Variant calling for the eight *Achromobacter xylosoxidans* whole genome sequences, see excel file attached, named: “Supplementary Table S1. Variant calling.xls”.

**Supplementary Methods**

**Assessment of phage activity by spot –test**

Spot-test was used to assess activity of the single phages (25 in total) from DSMZ collection and the phage cocktail APC 2.1 against *A. xylosoxidans* isolates. Hundred µl of overnight culture of each *A. xylosoxidans* isolate with concentration of 10^9^ CFU/ml was mixed with 3.5 ml of Tryptic soy (TS) broth (Becton Dickinson) supplemented with 0.6% agar at 45 °C and plated on Petri dishes with the bottom layer of TS agar (Becton Dickinson). Plates were air-dried for 10 min. Ten µl of each phage or/and phage cocktail and its 100-fold dilutions (up to 10^-6^) were spotted on the bacterial loans. Dilutions were spotted to see plaque-forming ability of the phages on the given bacterial isolates. Plates were incubated at 37 °C aerobically overnight. Results were visualized and recorded on the next day. The obtained lysis zones were defined as confluent lysis, semi-confluent lysis, opaque lysis and separate plaques zones based on degree of phage activity. An isolate was considered susceptible “S” if any the above mentioned type of lysis zone was observed. In case of negative result, an isolate was considered as resistant “R”.

**Whole genome sequencing**

Genomic DNA was obtained for each isolate by means of the High Pure PCR Template Preparation Kit (Roche). The DNA was fragmented using a Covaris ultrasonicator with settings for 500 bp fragments. Library construction was done using 600 ng of fragmented DNA with the NEBNext Ultra II kit (New England Biolabs), including 3 PCR cycles. Library fragments ranging from 550 to 1000 bp were recovered on a 2% E-gel (ThermoFisher Scientific). Finally, whole genome sequencing was carried out as a single-index paired-end 250 run on an Illumina MiSeq device.

***De novo* genome assembly and variant analysis**

Contaminating phiX-174 internal standard reads were removed using bowtie2 (v2.3.4) [1]. Adapter trimming was done using cutadapt (v1.15) [2]. A final quality check was done with FastQC (v0.11.7). *De novo* genome assembly was done using SPAdes (v3.13.2) [3] with standard settings for Illumina paired-end reads. This pipeline does read-error correction, contig assembly, as well as scaffolding and gap filling. Contigs smaller than 1000 nt were removed from the final assembly. Quality assessment of the assemblies was done with Quast (v5.0.2) [4]. The final genome assemblies were submitted to the European Nucleotide archive (ENA) as fasta files.

Digital DNA hybridization was carried out online with GGDC (v2.1) (<http://ggdc.dsmz.de/ggdc.php)> [5], and ANI was calculated with the stand-alone tool OAU v1.2 (<https://www.ezbiocloud.net/tools/orthoaniu>) [6], for all possible pairwise combinations of the *de novo* assembled genomes and the reference *Achromobacter* genomes.

Fast bacterial variant calling was done with the snippy pipeline (v4.4.5) (<https://github.com/tseemann/snippy>), using the sequencing reads and an annotated reference genome. To detect SNPs, this pipeline is using freebayes (v1.3.1) [7] with minimum read coverage of 10 and a variant call quality of 100.

Draft functional annotation of the *de novo* assembled genomes was obtained with Prokka (v1.13.7) [8].

**DNA extraction from BALs and strain-specific qPCR**

**DNA extraction**

The ‘High Pure PCR Template Preparation kit’ of Roche was used according to the manufacturer’s guidelines to extract DNA from the BAL samples.

**qPCR**

Two microliters of a tenfold dilution of the DNA extracts of the BALs were added to a total volume of 10 μl LightCycler 480 HRM master mix (Roche) containing 0.2 μM of each primer. Amplification was carried out on a LightCycler 480 (Roche), using the following program: pre-incubation for 5 min at 95 °C and amplification for 45 cycles of 10 s at 95 °C, 15 s at 60°C and 15 s at 72 °C, after which a high resolution melting curve was generated, using the following protocol: 1 min at 95 °C, 1 min at 40 °C, 1 s at 60 °C, followed by a gradual increase in temperature from 60 °C to 97 °C, using a ramp rate of 0.02 °C per second. qPCR results were analyzed using the standard LightCycler 480 Software, version 1.5 (Roche). Primer sequences are available upon request.

**Supplementary References**

1. Langmead, B.; Salzberg, S.L. Fast gapped-read alignment with Bowtie 2. *Nature Methods* **2012,** 9, 357-359.

2. Martin, M., Cutadapt removes adapter sequences from high-throughput sequencing reads. *EMBnet J* **2011,** 17,.**

3. Bankevich, A.; Nurk, S.; Antipov, D.; Gurevich, A.A.; Dvorkin, M.; Kulikov, A.S.; Lesin, V.M.; Nikolenko, S.I.; Pham, S.; Prjibelski, A.D.; Pyshkin, A.V.; Sirotkin, A.V.; Vyahhi, N.; Tesler, G.; Alekseyev, M.A.; Pevzner, P.A. SPAdes: a new genome assembly algorithm and its applications to single-cell sequencing. *J Comput Biol* **2012,** 19, 455-477.

4. Gurevich, A.; Saveliev, V.; Vyahhi, N.; Tesler, G. QUAST: quality assessment tool for genome assemblies. *Bioinformatics* **2013,** 29, 1072-1075.

5. Meier-Kolthoff, J.P.; Auch, A.F.; Klenk, H.P.; Göker, M. Genome sequence-based species delimitation with confidence intervals and improved distance functions. *BMC Bioinformatics* **2013,** 14, 60.

6. Lee, I.; Ouk Kim, Y.; Park, S.C.; Chun, J. OrthoANI: An improved algorithm and software for calculating average nucleotide identity. Int J Syst Evol Microbiol **2016,** 66, 1100-1103.

7. Garrison, E.; Marth, G., Haplotype-based variant detection from short-read sequencing. *arXiv preprint arXiv:1207.3907 [q-bio.GN]* **2012**.**

8. Seemann, T. Prokka: rapid prokaryotic genome annotation. *Bioinformatics* **2014,** 30, 2068-2069.
